# Proteome Analysis of the Wild and *YX-1* Male Sterile Mutant Anthers of Wolfberry (*Lycium barbarum* L.)

**DOI:** 10.1371/journal.pone.0041861

**Published:** 2012-07-30

**Authors:** Rui Zheng, Sijun Yue, Xiaoyan Xu, Jianyu Liu, Qing Xu, Xiaolin Wang, Lu Han, Deyue Yu

**Affiliations:** 1 National Key Laboratory of Crop Genetics and Germplasm Enhancement, National Center for Soybean Improvement, Nanjing Agricultural University, Nanjing, China; 2 College of Life Science, Ningxia University, Yinchuan, China; 3 Jiangsu Polytechnic College of Agriculture and Forestry, Jurong, China; Lawrence Berkeley National Laboratory, United States of America

## Abstract

Pollen development is disturbed in the early tetrad stage of the *YX-1* male sterile mutant of wolfberry (*Lycium barbarum* L.). The present study aimed to identify differentially expressed anther proteins and to reveal their possible roles in pollen development and male sterility. To address this question, the proteomes of the wild-type (WT) and *YX-1* mutant were compared. Approximately 1760 protein spots on two-dimensional differential gel electrophoresis (2D-DIGE) gels were detected. A number of proteins whose accumulation levels were altered in *YX-1* compared with WT were identified by mass spectrometry and the NCBInr and Viridiplantae EST databases. Proteins down-regulated in *YX-1* anthers include ascorbate peroxidase (APX), putative glutamine synthetase (GS), ATP synthase subunits, chalcone synthase (CHS), CHS-like, putative callose synthase catalytic subunit, cysteine protease, 5B protein, enoyl-ACP reductase, 14-3-3 protein and basic transcription factor 3 (BTF3). Meanwhile, activities of APX and GS, RNA expression levels of *apx* and *atp synthase beta subunit* were low in *YX-1* anthers which correlated with the expression of male sterility. In addition, several carbohydrate metabolism-related and photosynthesis-related enzymes were also present at lower levels in the mutant anthers. In contrast, 26S proteasome regulatory subunits, cysteine protease inhibitor, putative S-phase Kinase association Protein 1(SKP1), and aspartic protease, were expressed at higher levels in *YX-1* anthers relative to WT anthers. Regulation of wolfberry pollen development involves a complex network of differentially expressed genes. The present study lays the foundation for future investigations of gene function linked with wolfberry pollen development and male sterility.

## Introduction

In flowering plants, male reproductive processes take place in the stamen, a part of the anther, which contains diploid sporogenous cells that experience meiosis to form haploid microspores and finally develop into pollen grains or the male gametophyte [1]–[5]. Detailed analysis of anther development has shown that cell differentiation occurs in a precise chronological order, with distinct stages, which can be related to bud size [6]. Once this process becomes disordered, pollen production might be aborted, resulting in male sterility. Many genes controlling stamen and pollen development have been identified, and their specific roles characterized. These functions appear to be conserved in higher plants [7]–[11].

In recent years there has been an increasing application of proteomic approaches to study anther development and pollen reproduction, such as in Arabidopsis [12]–[15], rice [16]–[19], tomato [2], [20], and *Brassica napus* [21]–[23]. By applying proteomic analyses, many proteins specifically expressed in anthers with roles in pollen development [24], tapetum degradation [25], programmed cell death (PCD) [26], and callose hydrolyzation [27] were identified. Proteins involved in metabolic process, stress resistance [16], [28], and several transcription and translational regulating factors [29]–[31] were also characterized. It is difficult to find male sterile related genes affecting pollen development directly; therefore, methods for globally detecting different expression patterns between the male sterile mutant and the WT must be applied. The exploitation of plant male sterility is enhanced by combining transcriptome with proteome analysis of developing pollen [32]. In tomato, proteomic analysis of male-sterile *7B-1*mutant anthers at the stage when tetrads formed revealed that proteasome and 5B protein, with potential roles in tapetum degeneration, were down-regulated. Cystatin, regulator of endogenous proteolytic activities during seed maturation and germination and in PCD, were up-regulated and correlated with the male sterility [2]. In addition, proteins associated with carbohydrate and energy metabolism, photosynthesis and flavonoid synthesis were also down-regulated in CMS anthers of *Brassica napus*, all of which might have roles in pollen development [23]. Moreover, several proteins correlated with male sterility were identified in rice, with roles in protein synthesis, signal transduction, cell death and carbohydrate metabolism. The occurrence of male sterile mutants is usually explained by the lack of certain proteins/enzymes involved in pollen development. In the gametophytic male-sterile mutant *gaMS-2* of maize, reduced Zea m1 level is associated with the sterility [33]. GS was inactivated in tobacco anthers and microspores by a dominant-negative mutant approach, resulting in male sterility [34]. The conditional male sterile mutant of Arabidopsis was associated with the FLP1 protein, likely playing a role in the synthesis of the components of tryphine, sporopollenin of exine and the wax of stems and siliques. [35]. In addition, several housekeeping proteins with potential roles in microspore development also showed altered abundance. All of these identified proteins have important functions in pollen development in higher plants.

*Lycium barbarum* L, a woody bush, is a famous traditional Chinese herbal medicine that nourishes the kidneys and liver, brightens eyes, reduces blood glucose and serum lipids, and has anti-aging, immunomodulating, anticancer, anti-fatigue, and male fertility-facilitating properties [36], [37]. It has been widely used as a health food for 2300 years, with its fruit being used to produce various types of health products and foods, such as medicinal beverages and dietary soups [38], [39]. However, it is difficult to meet the need for substantial improvement of existing varieties of wolfberry by natural selection.

Plant male sterility is important for developmental and molecular studies and in hybrid seed programs [40]; therefore, many male sterile mutants in higher plants have been characterized. To the best of our knowledge, few studies on male sterility of wolfberry have been reported. The spontaneous male sterile mutant *YX-1* produces flowers with shrunken stamens, in which pollen production is aborted in early tetrad stage. *YX-1* mutants also show reduced stamen filament length relative to the WT, and appear beneath the receptive stigma at flower opening [41]. As the male sterile varieties are valuable resources that greatly facilitate the production of hybrids via cross-pollination, in-depth study of anther and pollen development, YX-1 male sterile mutant has important application value in hybrid breeding programs and important theoretical significance.

In this present study, we report the proteomes of the anthers of the WT and *YX-1* wolfberry and identify differentially expressed proteins (in terms of protein spot volume). We discuss their possible biological roles and their potential effects on anther development and pollen fertility, with the aim of understanding the molecular mechanism of the biological process at the proteomic level. The protein profiles of anthers at early tetrad stage, when tetrad and tapetum show normal development in WT anthers, but tapetum and tetrads degeneration are observed in *YX-1*, were compared by 2D-DIGE, and interesting proteins were identified by mass spectrometry. More than 1760 spots were observed on DIGE gels. Compared with the WT, in *YX-1* mutant anthers, proteins related to energy, carbohydrate and amino acid metabolism, pollen development, stress response, and signaling were down-regulated. In contrast, proteins involved in proteolysis were up-regulated. The possible functions of these proteins in pollen development and in male sterility are discussed. The results will provide important information for further studies on the molecular mechanism of male sterility in wolfberry.

## Materials and Methods

### Plant Growth and Anther Collection

The *YX-1* male sterile mutant and the WT were grown in the wolfberry orchard of Yinchuan Yuxin Wolfberry seed industry Co., Ltd., located in Yinchuan, Ningxia Municipality, China. Plants were watered regularly and fertilized weekly with a commercial fertilizer (40% Compound Fertilizer, Nantong Hailing Fertilizer Co., Ltd., Nantong, China).

**Figure 1:**
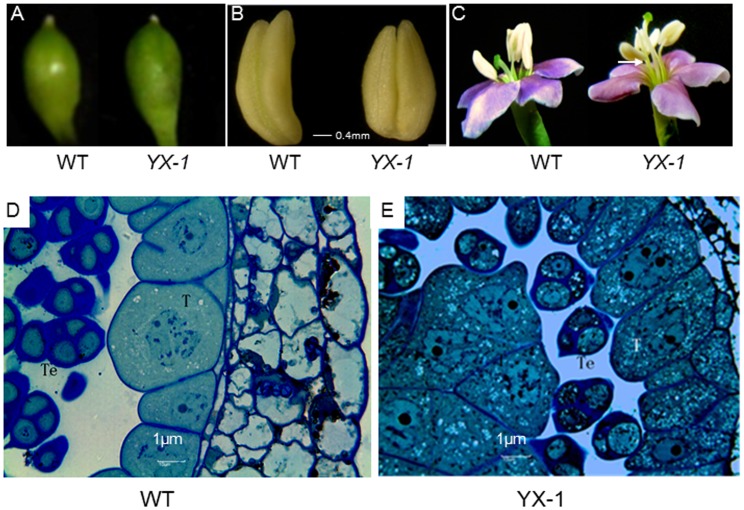
Morphological changes of *YX-1* mutant compared with WT about flower organs. A. Flower buds of WT and *YX-1* mutant of wolfberry. **B.** Stamens from WT and *YX-1* buds. **C.** Flowers of WT and *YX-1*, note the reduced stamen filament relative to the WT, appearing beneath the receptive stigma. **D.** Cross-section of WT anther from the same stage as in B, note the normal developed tapetum (T) and tetrads. **E.** Cross-section of *YX-1* anther from the same stage as in B, note the premature tapetum (T) and degraded tetrads (Te), both with masses of small bright vacuoles.

Flower buds of about 4.8–5.0 mm(WT)and 5.0–5.8 mm(*YX-1*)length (Fig. 1A, corresponding the early stage of tetrad) were picked using a combination of the bud size and anther cytological investigation, as previously reported [42], dissected under a microscope. Stamens from buds at the same stage,when tetrads and tapetum cell were degrading in *YX-1* anthers but showed normal development in WT anthers(confirmed by anther squashes, Fig. 1D)were isolated for 2D-DIGE analysis. Stamen samples were either used immediately or frozen in liquid nitrogen and stored at −80°C until further analysis.

### Protein Sample Preparation

Anther protein extractions were performed using a trichloroacetic acid (TCA)-acetone protocol [23] with some modifications. The samples of fresh or frozen wolfberry anthers were finely powdered in liquid nitrogen and homogenized with chilled acetone/10% TCA for 30 min, and then precipitated overnight at −20°C. Precipitated proteins were centrifuged at 12000×g for 45 min at 4°C. After three washes with acetone, the pellets were vacuum dried and proteins extracted with buffer containing 7 mol·L^–1^ urea, 2 mol·L^–1^ thiourea, 150 mmol·L^–1^ Tris-HCl (pH8.5), 4% CHAPS, and 1 mmol·L^–1 ^PMSF, by vortexing for 1 h at room temperature, and centrifugation at 12 000 g for 45 min at 4°C. The supernatants were collected and the protein content was determined according to the Bradford method, using the Bio-Rad protein assay reagent (Bio-Rad, Hercules, CA, USA), and samples were stored at −80°C until 2-DE.

### Fluorescence Labeling Protein with CyDyes

Protein labeling with fluorescent cyanine dyes was performed according to the manufacturer’s instructions (GE Amersham, Fairfield, CT, USA). Individual samples from three groups (pooled internal standard, *YX-1*, and the WT) were labeled with Cy2, Cy3, and Cy5, respectively. The three dyes were designed to ensure that proteins common to each sample have the same relative mobility regardless of the dye used to tag them. CyDyes were reconstituted in anhydrous DMF and combined with samples at a ratio of 400 pmol of CyDye to 50 µg of protein. Labeling was performed on ice and in the dark for 30 min. The reaction was then quenched by incubating with 1.5 µL of 10 mM lysine on ice in the dark for 10 min.

### 2-DE and Image Acquisition

Proteins were focused on 13 cm Immobiline Drystrips at pH3-10, with non-linear pH gradients (GE Amersham), using an Ettan IPGphor Isoelectric Focusing System (GE Amersham) for a total of 70 000 volt hours. After isoeletric focusing, IPG strips were equilibrated in buffer (6 M urea, 50 mM Tris-HCl, 30% glycerol, 2% SDS) supplemented with 1% DTT to maintain the proteins in a fully reduced state, followed by 3% iodoacetamide to prevent reoxidation of thiol during electrophoresis. Proteins were then separated on 12.5% SDS polyacrylamide gels using a Hofer SE 600 (GE Amersham). Samples were run in three biological replicates. The gels were scanned using a Typhoon FLA9000 (GE Amersham). Excitation/emission wavelengths for Cy2, Cy3, and Cy5 are 488/520, 532/580, and 633/670 nm, respectively.

### DIGE Analysis

Relative protein quantification across male sterile mutant *YX-1* and WT samples was performed using DeCyder 2-D Differential Analysis Software (v 6.05.11, GE Amersham). The Cy2-labeled pooled internal standard on every gel allowed accurate relative quantitation of protein spot features across different gels. The spots that were present on at least two gels based on the image analysis were considered as expressed protein spots. Student’s *t*-test (*p*<0.05) and one-way ANOVA were used to calculate significant differences in relative abundances of protein spot-features in the male sterile anther compared with the WT anthers. Spots with reproducible and significant variations, at least 1.5-fold up- or down-regulated, were considered differentially expressed proteins.

### In-gel Digestion

Protein spots were cut from gels, destained for 20 min in 30 mM potassium ferricyanide/100 mM sodium thiosulfate (1∶1 v/v), washed in Milli-Q water until the gels were colorless, and then lyophilized. Each spot was digested in 5 µL 10 ng/µL trypsin (sequencing-grade reagent, Promega, Fitchburg, WI, USA) at 37°C overnight. The peptides were extracted three times with 60% ACN/0.1% TFA. The extracts were dried completely by centrifugal lyophilization. The resulting tryptic digests were concentrated and desalted using C18 ZipTips (Millipore Corporation, Bedford, MA, USA) according to the manufacturer’s protocol.

### Mass Spectrometry and Data Analysis

Samples were mixed (1∶1 v/v) with 5 mg/ml HCCA matrix and analyzed by a 4800 Plus MALDI-TOF/TOF™ Analyzer (Applied Biosystems, Carlsbad, CA, USA). Calibration for MS/MS mode was carried out using fragment ion masses from Glufibrinopeptide. Initial analysis of protein digests on the 4800 instrument was carried out by MS in positive ion reflectron mode, using trypsin autolysis products (m/z 842.510, 1045.564 and/or 2211.105) for internal mass calibration where possible, or else the default calibration. Parent mass peaks with a mass range of 800–4000 Da and minimum S/N 50 were picked out for tandem TOF/TOF-MS/MS analysis. The 10 most abundant precursor ions in each spectrum (excluding trypsin autolysis fragments) were subsequently selected by Protein Pilot™ v4.0 software for MS/MS analysis, with collision induced dissociation (CID) closed, increased laser fluence, and 2500 laser shots collected per sample. The UV laser was operated at a 25 Hz repetition rate with a wavelength of 355 nm and the accelerated voltage was 2 kV.

Protein identifications were conducted by combining search (MS plus MS/MS) to the entries of a non-redundant protein and/or EST-viridiplantae database downloaded from the National Center for Biotechnology Information using MASCOT open source (http://www.matrixscience.com) in the NCBI non-redundant database. Search parameters were the enzyme trypsin, taxonomy restrictions to Viridiplantae, ±50 ppm peptide mass tolerance in MS, ±0.2 Da for MS/MS data, one missed cleavage, carbamidomethyl (C) as a fixed modification and methionine oxidation as a variable modification. The confidence in the peptide mass fingerprinting matches (*p*<0.05) was based on the MOWSE score and confirmed by the accurate overlapping of the matched peptides with the major peaks of the mass spectrum. Only significant hits, as defined by the MASCOT probability analysis (*p*<0.05), were accepted. The functional classification of the identified ESTs was performed following a BLAST search, and the protein sequences of matching ESTs were then searched against the NCBInr protein database. Only BLAST matches with E values ≤10^−30^ were selected [19].

### Quantitative Real-time RT-PCR Analysis

Quantitative real-time RT-PCR was used to assay gene expression levels for *apx* and *atp synthase beta subunit*. Primers for quantitative real-time RT-PCR analysis are shown in Table 1. Wolfberry actin (Accession number HQ415754.1) was used for RNA normalization. All wolfberry RNA samples were diluted to 200 ng µl^–1^. The SuperScript™ III platinum® two-step qRT-PCR kit with SYBR® Green (Invitrogen, Carlsbad, CA, USA) was used for detecting the expression levels of the genes. Quantitative real-time RT-PCR was carried out in a final volume of 20 µl containing 10 µl Platinum® SYBR® Green qPCR SuperMix-UDG, 10 µM forward and reverse primers in a 7500 Real time PCR System (Applied Biosystems). The relative expression levels of all the samples were analyzed according to recommendations in the User Bulletin for the 7500 Real time PCR System. All reactions were performed in three biological replicates. The threshold cycles (Ct value) of the target genes and actin in different samples were obtained by quantitative real-time RT-PCR.

**Table 1:** Specific primers used for quantitative real-time RT-PCR analysis.

Gene	Forward primer	Reverse primer
*apx*	5′-AACCTGAGCAATGCCCAGAA-3′	5′-TCATTTAGCCCCATCCTGTAGAA-3′
*atp synthase beta subunit*	5′-GGATCCGAAGTATCGGCCTTA-3′	5′-TGCGGGTACATAAACTGCTTGA-3′
*actin*	5′-GACCTTCAATGTTCCCGCTATG-3′	5′- GCCATCACCAGAGTCCAACAC-3′

### Enzyme Activity Assay

To monitor the activity of APX and GS, protein was extracted with 100 mmol L^−1^ sodium phosphate buffer (pH 7.0) containing 5 mmol L^−1^ ascorbate and 1 mmol L^−1^ EDTA, and 10 mmol L^−1^ Tris-HCl buffer (pH 7.6) containing 1 mmolL^−1^ MgCl_2_, 1 mmol L^−1^ EDTA and 1 mmol L^−1^ β-mercaptoethanol ), respectively from WT and mutant anthers. APX activity was determined by the decrease of absorbance at 290 nm (extinction coefficient 2.8 mM cm^−1^) as described by Chen and Asada [43].The reaction mixture was composed of 50 mM potassium phosphate buffer (pH 7.0), 0.5 mM ascorbate, 0.2 mM H_2_O_2_ and the appropriate volume of protein extract.

Determination of GS activity was performed by the method of Oaks *et al* [44]. The reaction mixture contained 80 mmol L^−1^ glutamate, 1 mmol L^−1^ hydroxylamine, 8 mmol L^−1^ ATP, 0.2 mol L^−1^ N-tris- (hydroxymethyl)methyl glycine (Tricine) (pH 7.8), 4 mmol L^−1^ MgSO_4_ and 0.2 mmol L^−1^ EDTA and the appropriate volume of protein extract, and the absorbance of the hydroxamate derivative of glutamic acid measured at 540 nm. One unit of GS activity was defined as 1 µmol L-glutamate γ-monohydroxamate formed per min. Protein in enzyme extract was estimated using Bradford method [45]. Samples were performed in three biological replicates.

## Results

### Anther Development

Under the growth conditions as mentioned in “Plant growth and anther collection”, buds at the early tetrad stage from *YX-1* mutant were longer (approximate 5.0–5.8 mm length) than that from the WT (approximate 4.8–5.0 mm length, Fig. 1A); and the stamens of the *YX-1* appeared less slender compared with those of the WT (Fig. 1B ). Also, *YX-1* mutants showed reduced stamen filament length relative to the WT, and appear beneath the receptive stigma at flower opening (Fig. 1C). Corresponding to the stage and bud lengths outlined in Fig. 1A the tapetal cells were intact, with a dense cytoplasm containing a few small vacuoles, both tetrads and tapetum showed normal development in WT anthers (Fig. 1D). In contrast, in *YX-1* anthers (Fig. 1E), tetrads and tapetal cells had started to degenerate, and numerous small vacuoles were present in the tapetum,also small vacuoles appeared in tetraspores. Anther squashes at an earlier stage (buds of 3.0–5.0 length mm) in both WT and *YX-1* showed tetrads enveloped within a thick callose wall, a rich cytoplasm in the tetraspores and tapetal cells radially enlarged with a dense cytoplasm. At later stages (buds of 6.0–7.0 mm length), microspores were observed in WT anthers, but in *YX-1* anthers, tetrads and the tapetal cells had completely disintegrated and disappeared, resulting in an empty anther chamber (not shown). This confirmed our earlier observation that in the *YX-1* mutant, pollen development breaks down at the early tetrad stage [42].

### 2D-DIGE Analysis of Anther Proteomes

**Figure 2:**
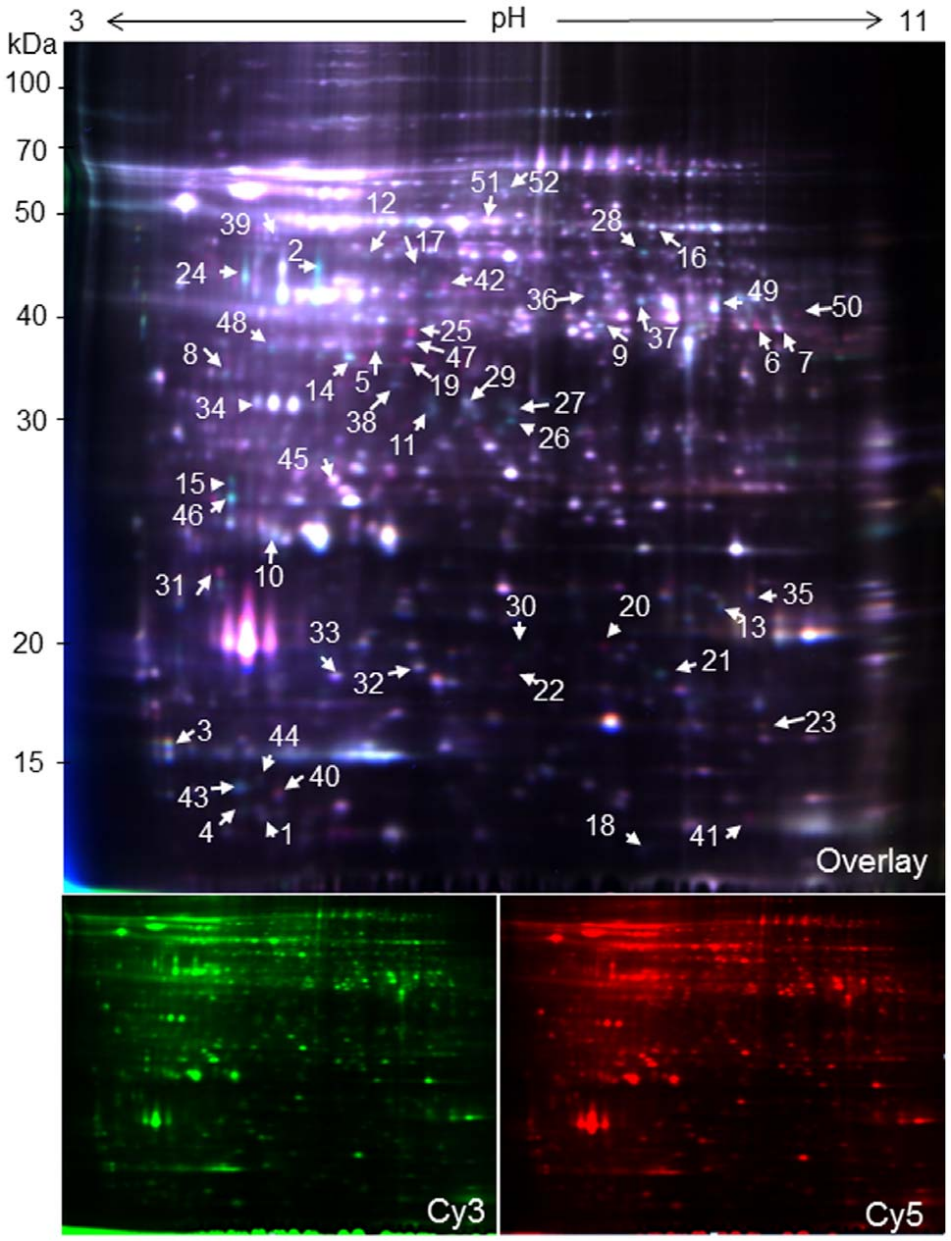
2D-DIGE images of anther proteins from the WT and male sterile mutant *YX-1* in wolfberry. Extracts from the WT and *YX-1* anther of three independent biological repeat experiments were differentially labeled with the spectrally resolvable CyDye fluors Cy3 and Cy5 and separated by two-dimensional electrophoresis (2-DE) on 13-cm (pH 3–11) IPG strips and 12.5% polyacrylamide gels. A merged image of Cy5-labeled *YX-1* (red) and Cy3-labeled WT (green) is shown. Arrowed and numbered spots in the image are differentially expressed protein spots. Molecular markers (in kDa) are shown on the left.

Soluble proteins of wolfberry anthers from *YX-1* (approximate 5.0–5.8 mm length) and the WT buds (approximate 4.8–5.0 mm length) were extracted. After preparative experiments, we selected pH 3–11 strips for proteome analysis. Due to the limitations of conventional 2-DE for reproducibility and sensitivity, we used the DIGE technology to study differentially expressed proteins in WT and *YX-1* anthers. Equal amounts (50 µg) of protein samples of WT and *YX-1* anthers were labeled with Cy2 (internal standard), Cy3, or Cy5 dyes. An overlay of the Cy3 and Cy5 images from the 2D-DIGE gels is shown in Fig. 2. The protein expression patterns of *YX-1* mutant were generally similar to those of WT anthers, and more than 1760 spots were observed by DIGE methodology.

**Figure 3:**
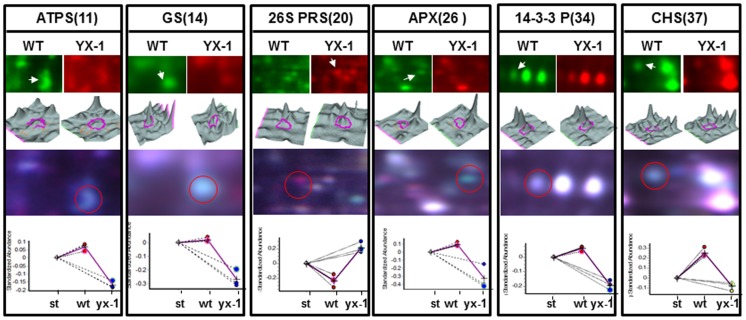
Analysis of several identified proteins. The readout of the DeCyder Biological Variation Analysis (BVA) module is shown for ATP synthase subunit E (ATPS, spot 11), putive glutamine synthetase (GS, spot No. 14), 26S proteasome regulatory subunit (26S PRS, spot No. 20), ascorbate peroxidase (APX, spot No. 26), 14-3-3 protein (14-3-3, spot No. 34) and chalcone synthase family protein (CHS, spot No. 37). Enlarged regions of 2D-DIGE gels for Cy3-labeled WT (green) and Cy5-labeled *YX-1* (red), and the corresponding 3D views, are represented. The bottom panel shows a graphic representation of the differences in abundance of these proteins across three independent experiments. For normalization purposes, a Cy2-labeled internal standard was included, corresponding to a pool of protein from all extracts used in the analysis (st, standard).

From the result of 2-DE image analysis, we found a number of spots with lower or higher protein abundances (measured as the relative spot volume) in *YX-1* anthers compared with those in the WT. A threshold limit of 1.5-fold was set in this study as previously reported[2] and three replicates were performed to reduce the number of potential false positives because of the sensitivity and reproducibility of DIGE technology. Fig. 2 shows a representative DIGE image of WT and *YX-1* anther protein extracts labeled with Cy3 and Cy5 and separated with IPG 3–11 strips and the numbered spots used for mass spectrometry analysis. Some of these differential spots are shown in the enlarged portion of gels in Fig. 3. Fifty-two spots showed at least a 1.5-fold change in protein abundance (*p*<0.05), among which, 13 showed an increase in *YX-1* mutant anthers and 39 showed decreased abundance compared with their levels in WT anthers.

### Protein Identification

For MALDI-TOF/TOF MS and MALDI-TOF/TOF MS/MS analysis, a total of 52 differently expressed spots with greater than 1.5-fold change in both genotypes (as marked in Fig. 2) were excised. Ultimately, 45 spots (86%) were successfully identified as 41 individual proteins by searching against NCBInr and Viridiplantae EST databases, proteins associated with pollen development and tapetal activities in other plants were found to be differentially expressed in *YX-1*, as shown in table 2. Four proteins were observed as multiple spots, e.g., three spots corresponding to ascorbate peroxidase (APX, with low levels in *YX-1* anthers), two spots corresponding to ribulose-1,5-bisphosphate carboxylase, two spots corresponding to glyceraldehyde-3-phosphate dehydrogenase (GAPDH) and two spots corresponding to copper chaperone. In WT anthers, proteins with higher expression, relative to the mutant, included putative glutamine synthetase (GS), ATP synthase subunits, malate dehydrogenase (MDH), plastid aldolase homolog, GAPDH, fructokinase-like protein, ribulose-1,5-bisphosphate carboxylase, APX, chalcone synthase (CHS), CHS-like protein, 5B protein, cysteine protease, protein disulfide isomerase, basic transcription factor 3(BTF3), calmodulin-like protein 1, 14-3-3 protein, putative callose synthase catalytic subunit, and enoyl-ACP reductase. In *YX-1* anthers, the up-regulated proteins included cysteine protease inhibitor 5, putative S-phase Kinase association Protein 1(SKP1), disulfide isomerase, 26S proteasome subunits, ubiquitin-protein ligase, and an aspartic protease.

**Table 2:** Proteins from WT and *YX-1* anthers of wolfberry analyzed by DIGE and MALDI-TOF/TOF, and identified by searching against NCBInr and Viridiplantae EST databases.

aSpot No.	bAcc. No.	cProtein name (Species)	dMr/pI	eScore	fS.C. (%)	gS.V.R. (YX-1/WT)	h *p*-value
**Photosynthesis-related**							
1	gi|170320	ribulose-1,5-bisphosphate carboxylase (NS)	10.3/5.37	100	80	−1.57	0.0083
2	gi|445628	RuBisCO activase(NT)	42.9/5.52	100	85	−2.76	0.0013
3	gi|230922	unactivated form Of ribulose-1,5- bisphosphatecarboxylase(NT)	14.7/5.19	100	80	−2.20	0.0048
4	gi|170320	ribulose-1,5-bisphosphate carboxylase (NS)	10.3/5.37	100	77	−1.70	0.00015
**Carbohydrate and energy metabolism**							
5	gi|1781348	homologous to plastidic aldolases (ST)	38.6/5.89	100	17	−1.65	0.0011
6	gi|4539543	glyceraldehyde-3-phosphate dehydrogenase (NT)	36.8/7.7	100	42	−2.52	0.00024
7	gi|4539543	glyceraldehyde-3-phosphate dehydrogenase (NT)	36.8/7.7	100	42	−2.38	0.0066
8	gi|21592495	fructokinase-like protein (AT)	35.2/5.12	100	27	−2.65	0.0027
9	gi|21388550	malate dehydrogenase(ST)	36.4/8.48	100	22	−1.88	0.0076
10	gi|48209968	ATP synthase D chain, mitochondrial (ST)	29.8/5.34	100	60	−2.61	0.0011
11	gi|9652289	putative ATP synthase subunit E (SL)	27.4/6.63	100	47	−2.73	0.00024
12	gi|56784991	putative ATP synthase beta subunit (OS)	45.9/5.33	100	22	−1.72	0.00078
**Nucleic acid metabolism**							
13	gi|8272416	nucleoside diphosphate kinase 3(BR)	21.5/7.98	100	36	−1.83	0.0012
**Amino acid metabolism**							
14	gi|28393681	putative glutamine synthetase(AT)	38.9/5.59	97.2	14	−2.97	0.00023
15	gi|18414289	ARD(AT)	23.5/4.99	99.9	14	−2.48	0.0033
16	gi|4049354	glycine hydroxymethyltransferase-like protein (AT)	50.9/8.13	100	22	−2.67	0.0052
**Protein metabolism**							
17	gi|19851	cysteine protease (NT)	40.8/6.0	96.2	26	−2.42	0.0017
18	gi|415833	5B protein (SL)	11.6/8.16	100	29	−1.95	0.00005
19	gi|37805883	putative S-phase Kinase association Protein 1(SKP1) (OS)	35.5/5.57	98.6	9	2.42	0.0039
20	gi|18424049	26S proteasome regulatory subunit (AT)	24.4/5.08	99.5	27	2.59	0.0054
21	gi|30693656	ubiquitin-protein ligase (AT)	16.8/6.2	100	46	−2.89	0.0024
*22	gi|309372357	NT2C-EST-0809 (NT) similar to aspartic protease gi|226503984(ZM)	18.7/6.64	99.9	10	2.23	0.011
23	gi|20137686	cysteine protease inhibitor 5(ST)	17.1/8.63	98	16	2.10	0.0052
24	gi|30692346	ribosomal protein S1(AT)	45.3/5.13	100	19	−2.88	0.00026
25	gi|1848212	protein disulfide-isomerase (NT)	40.1/5.99	100	14	2.74	0.0089
**Stress related**							
26	gi|34809902	ascorbate peroxidase(NT)	32.3/5.96	98.7	15	−2.76	0.0094
27	gi|34809902	ascorbate peroxidase(NT)	32.3/5.96	100	26	−1.53	0.0048
28	gi|21039134	ascorbate peroxidase (SL)	42.4/8.65	100	26	−2.44	0.0021
29	gi|55296784	putative peroxidase (OS)	35.4/6.43	95.1	25	−2.34	0.002
**Transcription factor**							
30	gi|15220876	putative transcription factor BTF3 (AT)	17.9/6.62	100	30	−2.17	0.00017
**Signaling-related**							
31	gi|9979177	translationally-controlled tumor protein(NT)	18.9/4.54	98.9	20	2.65	0.000038
32	gi|75319566	calmodulin-like protein 1(OS)	21.1/4.75	100	31	−1.77	0.006
33	gi|15233402	putative calcium-binding protein (AT)	21.2/4.59	100	40	−1.79	0.000012
34	gi|3766535	14-3-3 protein (ST)	29.4/4.78	100	58	−1.99	0.00035
**Anther development**							
35	gi|4588012	putative callose synthase catalytic subunit (GH)	21.9/8.42	100	27	−2.43	0.00067
36	gi|2326772	chalcone synthase -like protein(NS)	40.7/5.59	100	30	−2.59	0.0011
**Flavonoid synthesis**							
37	gi|15217605	chalcone synthase family protein (AT)	43.9/6.01	100	20	−2.78	0.0047
**Fatty acid synthesis**							
38	gi|2204236	enoyl-ACP reductase(NT)	33.9/6.41	99.9	8	−2.32	0.0003
**Unknown proteins**							
39	gi|115474835	Os08g0154300 (OS)	43.1/5.18	100	18	−1.87	0.0016
40	gi|2673909	hypothetical protein (AT)	13.1/4.59	99	18	2.24	0.00073
41	gi|54291158	hypothetical protein (OS)	14.0/7.7	99.8	21	2.43	0.0015
**Others**							
42	gi|56562181	formate dehydrogenase (SL)	42.4/6.87	100	30	2.54	0.00022
43	gi|15228869	copper chaperone (AT)	13.1/4.91	99.8	30	−2.96	0.0061
44	gi|15228869	copper chaperone (AT)	13.1/4.91	99.9	26	−1.50	0.0032
45	gi|30580343	caffeoyl-CoA O-methyltransferase 6(NT)	27.9/5.3	100	32	2.33	0.00022

a Spot number in 2-DE gel, as shown in Fig. 2.

b Accession number in NCBInr/EST database.

c Protein names and species from the NCBInr/EST database. NT, *Nicotiana tabacum;* NS, *Nicotiana sylvestris;* AT, *Arabidopsis thaliana;* SL, *Solanum lycopersicum;* ST, *Solanum tuberosum;* BR, *Brassica rapa;* ZM, *Zea mays;* OS, *Oryza sativa.*GH, *Gossypium hirsutum.*

d Theoretical molecular weight and pI of the identified proteins.

e Mascot protein score for ions complemented by the percentage of the confidence index (C.I.).

f Sequence Coverage.

g Spot volume ratios are the average for each spot from three replicate gels.

h Protein spots with a significant change in abundance (1.5-fold or above) between the WT and YX-1, and a P-value of≤0.05 were considered statistically significant.

* Spots from EST database.

Unidentified differently spots not listed.

**Figure 4:**
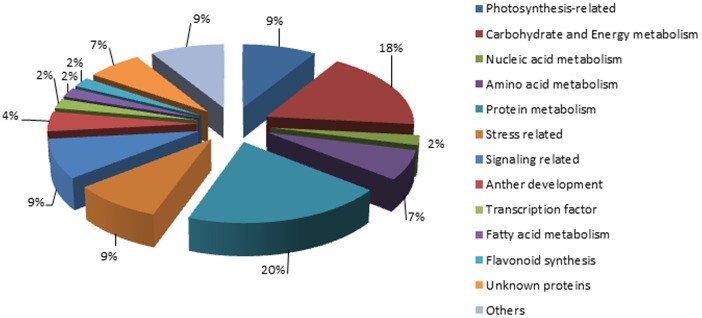
Function classifications of identified proteins in WT and *YX-1* anthers of wolfberry.

The 41 identified proteins could be classified into various functional groups (as shown in Fig. 4) based on the known functions from NCBI gene annotations and the literature. The largest numbers of protein spots with 1.5-fold or higher changes in abundance were those related to protein metabolism (20%), i.e., protein synthesis and folding, proteases and protease inhibitor(4 were up-regulated in WT and 5 in *YX-1* anthers), and carbohydrate and energy metabolism (18%, 8 highly expressed in WT anthers). Other groups of differentially expressed proteins were related to signaling (9%, 3 were up-regulated in WT and 1 in *YX-1* anthers), photosynthesis (9%, 4 highly expressed in WT anthers), stress response (9%, 1 were up-regulated in WT and 3 in *YX-1* anthers) and other proteins (9%). Relatively few proteins were classified into amino acid metabolism (7%), unknown proteins (7%), anther development (4%), transcription factor (2%), fatty acid metabolism (2%), nucleic acid metabolism (2%) and flavonoid synthesis (2%) groups (Fig. 4).

As can be seen from Table 2, between the WT and *YX-1* anthers, three differently expressed proteins were identified as mitochondrial ATP synthase related subunits (spots No. 10, 11 and 12), which are involved in energy metabolism. Three proteins correspond to APX (spots No. 26, 27, and 28), which is involved in stress response processes. GS (spot No. 15, Table 2) was one of the proteins that showed a major expression difference (Fig. 2 and 3) between the WT and *YX-1* anthers in DIGE gel images. Therefore, we selected these three enzymes to further confirm changes using enzyme activities or mRNA expression.

### Expression of apx and atp Synthase Beta Subunit mRNA

**Figure 5:**
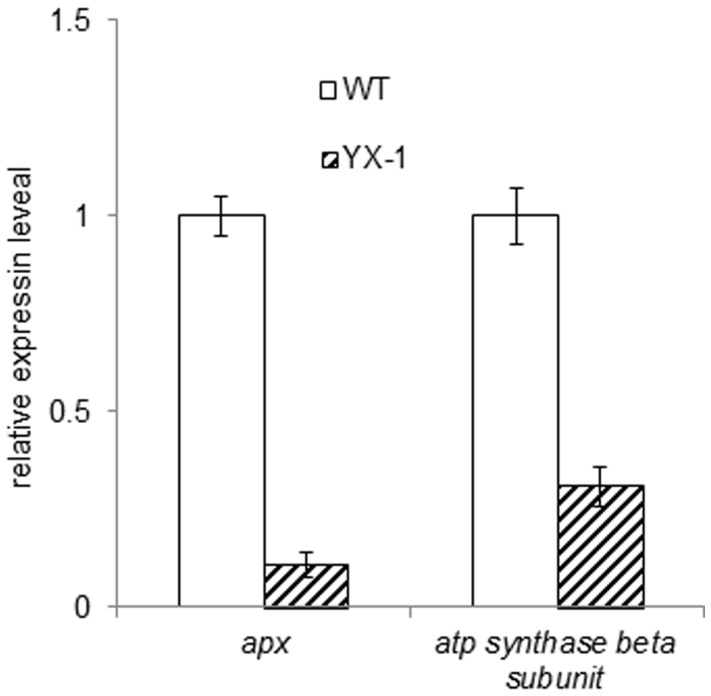
Quantitative real-time RT-PCR using SYBR Green assays for quantitative analysis of *apx* and *atp synthase beta subunit* mRNA expression levels in wolfberry anthers. Data are the mean ± SD from three replications.

To further understand the differential expressions of APX and component of the mitochondrial ATP synthase complex at the level of gene expression between the WT and *YX-1* anthers, quantitative real-time RT-PCR was used to analyze their expression levels at the stage of the early tetrad development (the same stage as the proteome analysis) between the male sterile anthers and the WT. It should be noted that these two genes had been isolated by 5′- and 3′-race methods in our previous experiments [46]. Fig. 5 shows the *apx* and *atp synthase beta subunit* mRNA expression levels in the wolfberry anthers. At the early tetrad stage of anther development, the expression level of *apx* and *atp synthase beta subunit* mRNA in *YX-1* anthers was only 11% and 31% to that in WT anthers, respectively. The results clearly indicated that expression of *apx* and *atp synthase beta subunit* mRNA is significantly reduced during the process of anthers abortion.

### Enzyme Activity

**Figure 6:**
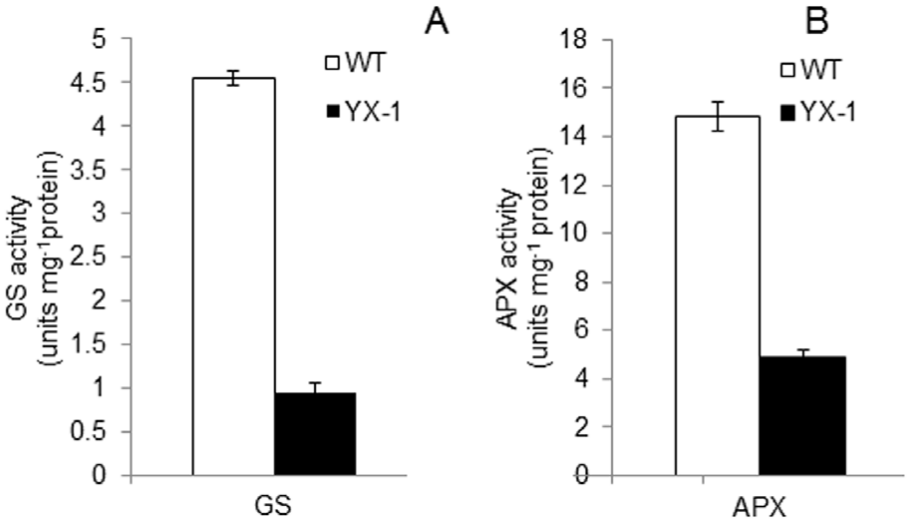
The activities of glutamine synthetase (GS) (A) and ascorbate peroxidase (APX) (B) in WT and *YX-1* anthers (of the same stage as shown in Fig. 1B). Error bars indicate standard deviation.

GS (spot No. 14) showed maximum difference in expression between the WT and the mutant in DIGE gel images (Fig. 2); in addition, three spots (spot No. 26, 27 and 28) were identified to be APX, which displayed low abundance in mutant anthers. Therefore, we analyzed the activity of these two enzymes in WT and *YX-1* anthers. The activity of GS was nearly 5 times, and that of APX approximately 3 times, higher in WT compared with that in *YX-1* anthers (Fig. 6A and 6B), which correlated with the 2-D DIGE results.

## Discussion

There has been little detailed proteomic characterization of the male sterile wolfberry. Therefore, we conducted a comprehensive proteomics analysis between mutant *YX-1* and WT anthers to gain an understanding of the mechanisms of wolfberry male sterility. The potential roles of some differentially expressed proteins in anther development and pollen fertility are discussed below.

The tapetum is universally present in higher plant anthers. In addition to its main function of supplying nutrition for meiocytes/spores, the tapetum has other roles, such as the production of the locular fluid, callase, pollenkitt/tryphine, sporophytic proteins and enzymes, and the formation of exine precursors, all of which are required for the normal development of microspores to pollen grains [4]. The tapetum undergoes cellular degradation during the late stage of anther development, which is considered a programmed cell death (PCD) event. Furthermore, tapetal cell disintegration accords well with the post-meiotic anther development processes. A premature or delayed degradation of the tapetum can result in male sterility [47]. In normal anthers of wolfberry, both tetrads and tapetum show normal development, with a dense cytoplasm and only a few small vacuoles present at the early tetrad stage (the stage used in the present study, Fig. 1D). In contrast, in the *YX-1* mutant anthers at the same stage, the tapetum begins to degenerate, coupled to numerous small vacuoles which also appear in tetraspore (Fig. 1E), which suggests that premature tapetum degeneration in *YX-1* could contribute to male sterility.

A number of enzymes, belonging to the carbohydrate and energy metabolism group, are reduced in *YX-1* anthers, relative to the WT. These down-regulated enzymes include mitochondrial ATP synthase subunits, fructokinase-like protein, MDH, aldolase, and GAPDH (Fig. 2 and Table 2). In CMS lines of rice and *B. napus*, some of the enzymes involved in energy and carbohydrate metabolism are also down-regulated [16], [23]. In particular, three protein spots were identified to be mitochondrial ATP synthase D chain (spot No.10, Fig. 2 and Table 2), putative ATP synthase subunit E (spot No.11, Fig. 2, 3 and Table 2), and mitochondrial ATP synthase beta subunit (spot No.12, Fig. 2 and Table 2). Cytoplasmic male sterility in plants is generally the consequence of dysfunction of mitochondria in the pollen, and several mitochondrion DNA regions encoding F0F1-ATPase (i.e. ATP synthase) subunits have been identified associated with CMS [48]. Plant mitochondrial genomes contain approximately 60 open reading frames (ORFs), among which, orf25 is implicated playing a role in the CMS of T cytoplasm maize [49], and orfB is involved in CMS in several plant species [50]–[52]. An accepted hypothesis on the mechanism of CMS is that the increased demand for respiratory function and cellular energy in the form of ATP during anther development may be compromised by expression of the aberrant mitochondria genes. ATP synthase β-subunit was observed in pollen mitochondria and was found to be generally important for male gametophytic development [53]. If the β-subunit is defective, it will cause the dysfunction of F0F1-ATPase, which may impact the energy output of mitochondria, resulting in abnormal anther development with non-functional pollens [48], [54], [55]. In the present study, protein spot No.12 was identified as beta subunit of ATP synthase F1 sector and it is inferred to be a defective protein, which may lead to the dysfunction of F0F1- ATPase by incorporation into the ATP synthase complex. Quantitative real-time RT-PCR showed that the expression of *atp synthase beta subunit* RNA was reduced by approximately 70% in *YX-1* anthers, relative to the WT (Fig. 5), which is consistent with the difference in protein abundance. In fact, it has been suggested that during microspore development, demand for energy is especially high. The reduced level of ATP synthase beta subunit suggests that the male sterile mutant plants are in an energy starved state. This is in accordance with down-regulation of genes controlling enzymes associated with energy in a CMS line of *B. napus* [23], [56].

Four down-regulated proteins are involved in carbohydrate metabolism: fructokinase-like protein, MDH, aldolase, and GAPDH. Starch is synthesized in anthers before meiosis and subsequently hydrolyzed to provide energy for lipid synthesis in both tapetum and microspores [57]. Decreased abundance of these enzymes in *YX-1* anthers could alter levels of sugar and starch, two molecules key to biosynthesis and energy balance.

Several other proteins/enzymes, including GS, APX, putative callose synthase catalytic subunit, CHS, CHS-like and enoyl-ACP reductase, were down-regulated in *YX-1* anthers, and these proteins/enzymes may have a role in tapetum and pollen development. One of the most notable difference in WT and *YX-1* anther gels was the location of the protein putative GS (spot No.14, Fig. 2, 3, and Table 2), and may be involved in pollen development. GS is found as two isoforms, cytoplasmic GS1 and chloroplastic GS2, catalyzing the ATP-dependent conversion of glutamine to glutamate. Isolated and in vitro-cultured microspores were unable to develop into functional pollen grains in a medium lacking glutamine [58], which indicated that glutamine plays a key role in plant amino acid metabolism and pollen development. The importance of GS1 in pollen reproduction has been shown in rice [59] and maize [60]. In tobacco, GS1 was inhibited by introducing mutated tobacco GS genes fused to the tapetum-specific TA29 and microspore-specific NTM19 promoters, and pollen aborted close to the first pollen mitosis in the transgenic plants, resulting in male sterility [61]. *YX-1* anthers also showed lower GS activity relative to WT (Fig. 6A). The decreased GS activity in *YX-1* anthers could cause a reduction in glutamine, which is required for pollen development thereby resulting male sterility.

It is notable that three spots (spot No.26, 27 and 28; Fig. 2 and Table 2) were identified as APXs and all of them showed lower amounts in *YX-1* relative to WT anthers. Just as in cotton and rice [62], [63], lower activity/amounts of oxidative stress enzymes in cytoplasmic male-sterile anthers was detected compared with fertile anthers. Moreover, the expression of *apx* RNA and the APX activity were lower in *YX-1* anthers, respectively, relative to the WT (Fig. 5 and Fig. 6B), during the process of anther abortion, when a great deal of ROS might be generated in the anther cell.

Callose synthase is responsible for the synthesis of callose deposited at the primary cell wall of meiocytes, tetrads and microspores in Arabidopsis, and T-DNA insertion mutations of the *CalS5* gene resulted in degeneration of microspores, thereby, male sterility [64]. Several studies have also described mutations in callose wall formation and dissolution in petunia [65] and tobacco [66] that disrupt fertility. Putative callose synthase catalytic subunit (spot No.35; Fig. 2 and Table 2) showed low spot volume in *YX-1* anthers. Collectively, the evidence indicates that the timing of callose formation and dissolution are critical for normal fertility.

Most plant phenolics, including flavonoids, are products of phenylpropanoid metabolism. CHS is one of the main enzymes in the flavonoid biosynthesis pathway, and an alteration in CHS abundance would be expected to affect the accumulation of all classes of phenolic compounds. Generally, tapetal cells produce proteins and lipids, as well as flavonoids, which are secreted into the pollen sac and form part of the exine [4]. Several enzymes involved in secondary metabolism, including CHS, are specifically or predominantly expressed in the tapetum [67]. It is reported that CHS is essential for pollen development and fertility in several plant species, and disruptions to CHS activity in the anthers resulted in the production of sterile pollen [68]–[72]. In addition, in recent research in Arabidopsis anthers, *LAP5* and *LAP6*, encoding anther-specific proteins with similarity to CHS, were suggested to play a role in pollen development and exine formation [73]. All of the above results suggest that flavonoids play an important role in the development of functional pollen. This study showed that two proteins, CHS (spot No.37, Fig. 2, 3, and Table 2) and CHS-like protein (spot 36, Fig. 2 and Table 2) are down-regulated in the mutant, indicating the premature degradation of the tapetum in *YX-1* mutant is concomitant with the reduction of anther specific CHS abundance. Thus, the level of flavonoids might decrease to below the level required to generate the pollen exine, leading to male sterility.

As a catalytic component of the fatty acid synthetase system in plants, enoyl-ACP reductase is prominently expressed in the tapetum, developing pollen grains, and vascular tissue of anthers. In the Arabidopsis *mod1* mutant, reduced activity of enoyl-ACP reductase led to abnormal development of various organs and reduced fertility [74]. It is also reported that the *DPW* gene, encoding a fatty-ACP reductase, is expressed in both tapetal cells and microspores during anther development in rice, and in a *dpw* mutant, defective anther development and degenerated pollen grains with an irregular exine appeared [75]. In *YX-1*, a protein identified as enoyl-ACP reductase (spot No. 38, Fig. 2, and Table 2) showed reduced abundance, which might affect fatty acid synthesis and anther development.

Besides metabolic pathways, proper anther development requires diverse regulatory processes. 14-3-3 proteins, being conserved phosphopeptide binding proteins in eukaryotic organisms [76], [77], regulate diverse biological processes in plants, such as metabolism, transcription, organellar protein trafficking, and stress responses [78], [79]. There have been some reports that 14-3-3 proteins are associated with ATP synthases in a phosphorylation-dependent style, playing a regulatory role in starch accumulation [80], regulation of PCD as a MAPKKKa-interacting protein in pollen development [81], In maize, reduced abundance of the14-3-3 protein led to temporal gene expression changes and, ultimately, pollen sterility [31]. In *YX-1* anthers, a 14-3-3 protein (spot No.34, Fig. 2, 3, and Table 2) abundance was down-regulated compared with the WT. The aberrant abundances of such 14-3-3 factors could contribute directly to *YX-1* defects. Another protein, BTF3 (spot No.30, Fig. 2 and Table 2) was also detected as having a reduced abundance level in *YX-1* anthers relative to the WT. BTF3 is the β-subunit of the nascent-polypeptide-associated complex, with a conserved role in regulating protein localization during translation in plants [82]. In a photoperiod-sensitive male-sterile mutant of rice, defects in pollen development were related to abnormal protein localization in anther tissue layers, including the tapetum [83]. The reduced abundance of BTF3 in male sterile anthers was similar to results obtained in tomato [2], which was considered to affect protein localization in the anther and hence affect pollen development.

In *YX-1* anthers at the early tetrad stage, some of proteolytic enzymes, including aspartic protease, 26S proteasome regulatory subunit and SKP1,as well as cysteine protease inhibitor, were up-regulated and these proteins may have a role in tapetum degeneration. Aspartic protease acts as an anti-cell-death factor participating in PCD, and overexpression of the gene encoding aspartic protease resulted in male sterility in Arabidopsis [84]. In common with the observation in the 7B-1 male sterile mutant of tomato [2], spot No.22 (Fig. 2 and Table 2) was identified as aspartic protease with increased amounts in mutant anther. The higher abundance of aspartic protease in the *YX-1* anther could disturb PCD of the tapetum and pollen development, causing male sterility.

Another protein with higher spot volume in *YX-1*, relative to WT anthers, was 26S proteasome regulatory subunit (spot No.20, Fig. 2, 3 and Table 2). Proteasomes are regulators of many processes such as the cell cycle, embryogenesis, metabolism, gametophyte survival, hormone signaling, senescence and defense [85], [86], and have been identified in plant reproductive organs, such as anthers [2], [86]. During PCD, proteasomes are released into the extracellular space and have the potential to damage nearby cells. The higher level of this protein in *YX-1* compared with WT anthers might disturb the degradation of regulatory proteins in anther tissues, hence leading to premature degradation of the tapetum and male sterility. Selective proteolysis of proteins mediated by the ubiquitin pathway is an important pathway for controlling many biological events. The SCF class of E3 ubiquitin ligases controls the ubiquitination of a wide variety of substrates, thereby mediating their degradation by the 26S proteasome. In Arabidopsis, it was reported that the Skp1 homologue ASK1 involved in the regulation of pollen development, and the *ask1-1* mutant produces polyads containing microspores of variable number and size, leading to non-viable pollen grains and male sterility [87]. In this study, the reduced level of protein spot putative SKP1 (spot No.19; Fig. 2 and Table 2) in *YX-1* anthers may affect pollen development.

In plants, cysteine protease inhibitor act as regulators of endogenous proteolytic activities. In rice, the TDR gene controls tapetum degeneration by targeting anther specific cysteine protease and protease inhibitor genes [88]. The male sterile mutant showed a reduced activity of cysteine protease (spot No.17; Fig. 2 and Table 2) but higher activity of cysteine inhibitor (spot No.23; Fig. 2 and Table 2) and this would inhibit cysteine protease activity and disturb tapetum development thereby effecting male sterility.

Other proteins were also reduced in *YX-1* anthers, such as 5B protein, considered to be related to tapetum degradation by inhibiting proteasome activity, are cysteine-rich and are specifically expressed in the tapetum and stamen in plants [25], [89]. In tomato, the 5B protein showed lower abundance at the tetrad stage in male sterile mutant 7B-1 [2]. In our study, the abundance of the 5B protein (spot No.18, Fig. 2 and Table 2) was reduced in the *YX-1* anther relative to the WT, which indicated that tapetum development was disturbed by abnormal abundance of the 5B protein, leading to pollen abortion. In addition, a number of other proteins showed altered abundance, including calmodulin-like protein 1 (spot No.32; Fig. 2 and Table 2) and putative calcium-binding protein(spot No.33; Fig. 2 and Table 2), which translates a signal of cytosolic Ca^2+^ elevation to downstream protein targets in numerous signal transduction cascades [90]. The altered abundance level of these enzymes/proteins in *YX-1* anthers might affect the abundance of regulatory proteins in anther tissues and, ultimately, pollen development.

### Conclusions

This study applied a proteomic approach to identify regulating proteins in the anthers of a male sterile mutant of wolfberry. We conclude that the breakdown of pollen development at the early tetrad stage of *YX-1* mutant anthers is associated with the differential expression of several proteins, including energy conversion related (e. g., ATP synthase subunits), amino acid metabolism related (e.g., GS), stress response related (e.g., APX), proteins with roles in signaling (e.g., 14-3-3 protein), anther development (e.g., putative callose synthase catalytic subunit), as well as proteases and protease inhibitor (e.g., 5B protein, 26S proteasome regulatory subunits, aspartic protease, cysteine protease, cysteine protease inhibitor and putative SKP1). Significantly, the abnormal protein complex of ATP synthase may cause the dysfunction of mitochondrion, and alterations of some protein abundances (such as APX, GS) could be the consequences of mitochondrial dysfunction in the pollen as indicated by changes in abundance of subunits of ATP synthase. These data indicate regulating patterns of wolfberry pollen development is a complex network. The significant impact on pollen fertility in *YX-1* appears to be the result of regulation of multiple metabolic pathways with differentially expressed genes potentially involved, which is supported by the results in male-sterile mutants of Arabidopsis, rice, tomato and maize [2], [32], [91], [92].
